# From cigarettes to compulsions: a longitudinal study in de novo Parkinson's disease

**DOI:** 10.3389/fpsyg.2025.1708535

**Published:** 2025-12-17

**Authors:** Matilde Massara, Luca Vedovelli, Fabio Masina, Nicky M.J. Edelstyn, Patrizia Silvia Bisiacchi, Elisa Di Rosa

**Affiliations:** 1 Department of General Psychology, University of Padova, Padova, Italy; 2 Unit of Biostatistics, Epidemiology, and Public Health, Department of Cardiac, Thoracic, Vascular Sciences and Public Health, University of Padova, Padova, Italy; 3 IRCCS San Camillo Hospital, Venice, Italy; 4 Department of Psychology, Bath Spa University, Bath, United Kingdom

**Keywords:** cigarette smoking, Parkinson's disease, Cognition, impulsivity, depression

## Abstract

**Introduction:**

Parkinson's disease (PD) is the second most common neurodegenerative disorder after Alzheimer's disease. Among the environmental and lifestyle factors associated with disease onset, cigarette smoking represents one of the most paradoxical. While substantial evidence has demonstrated a protective effect of smoking against the development of PD, smoking appears to worsen symptomatology, particularly by exacerbating impulsive-compulsive behaviors (ICBs) in people with PD (PwPD). However, longitudinal studies examining the effects of cigarette smoking on the progression of PD remain limited. Moreover, recent studies often involve mixed samples of treated and untreated PwPD, potentially confounding the impact of dopamine replacement therapy with that of smoking on ICBs.

**Methods:**

In the present study, we investigated a cohort of de novo PwPD, tracking their motor, cognitive, affective, and behavioral outcomes over 5 years, to better clarify the role of smoking in disease progression. Data were obtained from the Parkinson's Progression Markers Initiative and included 166 PwPD (119 non-smokers and 47 former smokers) and 79 healthy controls (48 non-smokers and 31 former smokers).

**Results:**

Our results revealed that a significantly higher percentage of former-smoker PwPD (28%) exhibited at least one ICB compared to non-smoker PwPD (13%; Pearson's ^2^(1) = 5.45, *p* = 0.02). No other significant differences between non-smokers and former smokers emerged in motor or non-motor symptoms, either in PwPD or in healthy individuals.

**Discussion:**

In conclusion, the novelty of our findings lies in showing that smoking-related influences on impulsive-compulsive behaviors in PD are most evident at the de novo stage, before any dopaminergic treatment. This temporal specificity may help resolve previous inconsistencies in the literature and underscores the importance of distinguishing between environmental and pharmacological effects on symptom development.

## 1 Introduction

Parkinson's disease (PD) is a major and rapidly growing public health challenge, ranking as the second most common neurodegenerative disorder after Alzheimer's disease (Poewe et al., 2017). Affecting approximately 1% of individuals over 60, PD manifests through hallmark motor symptoms, bradykinesia, rigidity, resting tremor, and postural instability. Still, its complexity extends to cognitive, behavioral, and affective impairments that profoundly impact People with PD (PwPD) quality of life. These non-motor symptoms can interfere with multiple domains of daily functioning, including the ability to manage personal finances, maintain employment, and adhere to legal and social responsibilities. For example, affective disorders frequently observed in PwPD, such as depression, can further compromise cognitive and financial capacities, especially among individuals with dementia (Giannouli and Tsolaki, 2019). Similarly, apathy, depression, and slowed information processing interfere with instrumental activities of daily living such as driving, medication management, and household organization. These functional consequences not only increase caregiver dependency but also carry significant socioeconomic implications for PwPD and their families (Martinez-Martin et al., 2011). Despite extensive research, disease-modifying treatments remain elusive, making symptom management the primary focus of clinical care.

While heritable forms of PD account for only 4–5% of cases, with mutations in LRRK2, PARK7, PINK1, and SNCA contributing to familial risk, most cases are sporadic and show only modest heritability (h^2^ ≈ 0.19) (Keller et al., 2012; Nalls et al., 2019; Wirdefeldt et al., 2011). This limited genetic contribution highlights the importance of other factors, particularly environmental exposures and modifiable lifestyle influences, in shaping disease onset, progression, and therapeutic response. Understanding how these factors interact with genetic vulnerability remains a central question in PD research aimed at improving PwPD outcomes.

Among these factors, tobacco smoking presents a paradox. Decades of epidemiological data show that smoking is associated with a reduced risk of developing PD (Chen et al., 2021; Hernán et al., 2002; Mappin-Kasirer et al., 2020). In a cohort of over 30,000 British male physicians, Mappin-Kasirer et al. (2020) reported that current smokers had a 30% lower risk of PD (RR = 0.71), while persistent smokers had a 40% lower risk (RR = 0.60). This inverse association is dose-dependent and reversible, suggesting an active neuroprotective or symptomatic-masking effect (Noyce et al., 2012).

Mechanistically, constituents of cigarette smoke, including nicotine, phenols, and phenolic acids, modulate dopaminergic signaling by inhibiting MAO-B and COMT, reducing dopamine reuptake, and suppressing α-synuclein aggregation and neuroinflammation (Omaye, 2002; Quik et al., 2008; Rose et al., 2024; Ruan et al., 2023). These effects may induce neuroplastic changes that enhance synaptic dopamine levels in the dorsal striatum, possibly by supporting a “dopamine reserve” that delays the onset of motor symptoms.

While these neurochemical effects may delay the onset of motor symptoms, they may also initiate long-term changes in the brain's reward circuitry, which could have detrimental consequences for incentive-driven decision-making. Chronic nicotine exposure may also induce sensitization of the mesolimbic dopamine system, particularly within the ventral striatum, through mechanisms such as upregulation of nicotinic receptors, altered dopamine transporter (DAT) expression, and modifications in D2/D3 receptor signaling (Mansvelder and McGehee, 2002; Robinson and Berridge, 2008). This sensitization primes the reward pathways once dopamine replacement therapy (DRT) is initiated, potentially increasing vulnerability to impulsive compulsive behaviors (ICBs), including pathological gambling, compulsive shopping, hypersexuality, and binge eating (Carbone and Djamshidian, 2024; Valença et al., 2013). In this way, smoking may paradoxically increase the risk of specific nonmotor complications in PD, even as it appears to delay the classic motor features.

Behavioral evidence linking smoking to ICB in PD remains mixed but generally suggests an increased risk associated with tobacco use. Weintraub et al. (2010) conducted an extensive cross-sectional study involving 3,090 PwPD from seven US movement disorder centers (mean age ~ 64 years; 63% male), covering various disease stages. They reported that current smokers had a modestly increased odds of exhibiting ICBs (OR = 1.22, 95% CI: 1.08–1.38) after adjusting for confounders such as age, sex, disease duration, and dopamine agonist use. Similarly, Callesen et al. (2014) examined 490 Danish PwPD (mean age ~65; 62% male) in a retrospective cohort and found a stronger association between current smoking and ICBs (OR = 3.44, 95% CI: 2.08–3.54), also highlighting the role of psychological traits in mediating this risk. Meta-analyses like Molde et al. (2018), which pooled data from multiple multinational cohorts, corroborate increased impulsivity and neuropsychiatric symptoms in PwPD with ICBs. However, smoking was a secondary factor in these analyses.

More recently, Zhu et al. (2023) employed a longitudinal design to investigate the relationship between smoking and impulse control behaviors in PD. Their cohort comprised 401 PD patients recruited from the Parkinson Progression Marker Initiative (PPMI) database (Marek et al., 2018), including both de novo (newly diagnosed, untreated) and medicated individuals at baseline, alongside 185 matched healthy controls. The PD group had a mean age of approximately 66 years and included roughly equal numbers of males and females. At baseline, disease-specific clinical measures indicated a median disease duration of approximately 1.5 years. Motor severity was assessed by the Unified Parkinson's Disease Rating Scale (Goetz et al., 2008; Hoehn and Yahr, 1967) Part III, which scored an average of 22 points, and Hoehn and Yahr staging was mostly 1–2, indicating early to moderate disease stages. Participants were followed over 5 years, allowing assessment of ICB development and progression relative to smoking status and dopaminergic treatment. While the study provided valuable longitudinal data suggesting smoking may influence ICB trajectories, the mixed composition of the PD cohort, combining untreated and treated PwPD, posed a significant limitation. Because DRT itself modulates ICB risk, the inclusion of both untreated and treated PwPD at baseline presents a substantial challenge in isolating the direct effects of smoking, potentially confounding results and limiting causal interpretation.

This heterogeneity highlights a significant limitation in previous research, which we address by focusing exclusively on a well-characterized cohort of de novo PwPD, tracking their motor, cognitive, affective and behavioral outcomes longitudinally over 5 years to clarify smoking's role in disease progression better.

Our “sensitization model” generates testable predictions. Specifically, in the PwPD, we predict that smokers, compared to non-smokers, will:

- present with milder motor symptoms at diagnosis,
- demonstrate slower motor decline
- develop ICBs and affective symptoms more rapidly and severely
- exhibit faster cognitive decline over the 5 years.

## 2 Materials and methods

### 2.1 Participants

Data used in the preparation of this article was obtained in April 2022 from the Parkinson's Progression Markers Initiative (PPMI) database (www.ppmi-info.org/access-dataspecimens/download-data), RRID:SCR_006431. For up-to-date information on the study, visit www.ppmi-info.org. PPMI is an observational, multicenter study designed to enhance research on PD progression through longitudinal follow-ups in a large participant sample. The included data were obtained from the PPMI Online Protocol (Tanner and Marek, 2025) and from the Followup of Persons with Neurodegenerative Diseases (FOUND) in the PPMI protocol, which collects information on the lifestyles of a subgroup of participants of the PPMI database. The FOUND project, initiated by the University of California, San Francisco, comprises various questionnaires on habits and lifestyle that can be completed online at the provided link (https://redcap.ucsf.edu/surveys/?s=ENCEM47ALY988KW4).

**Figure 1:**
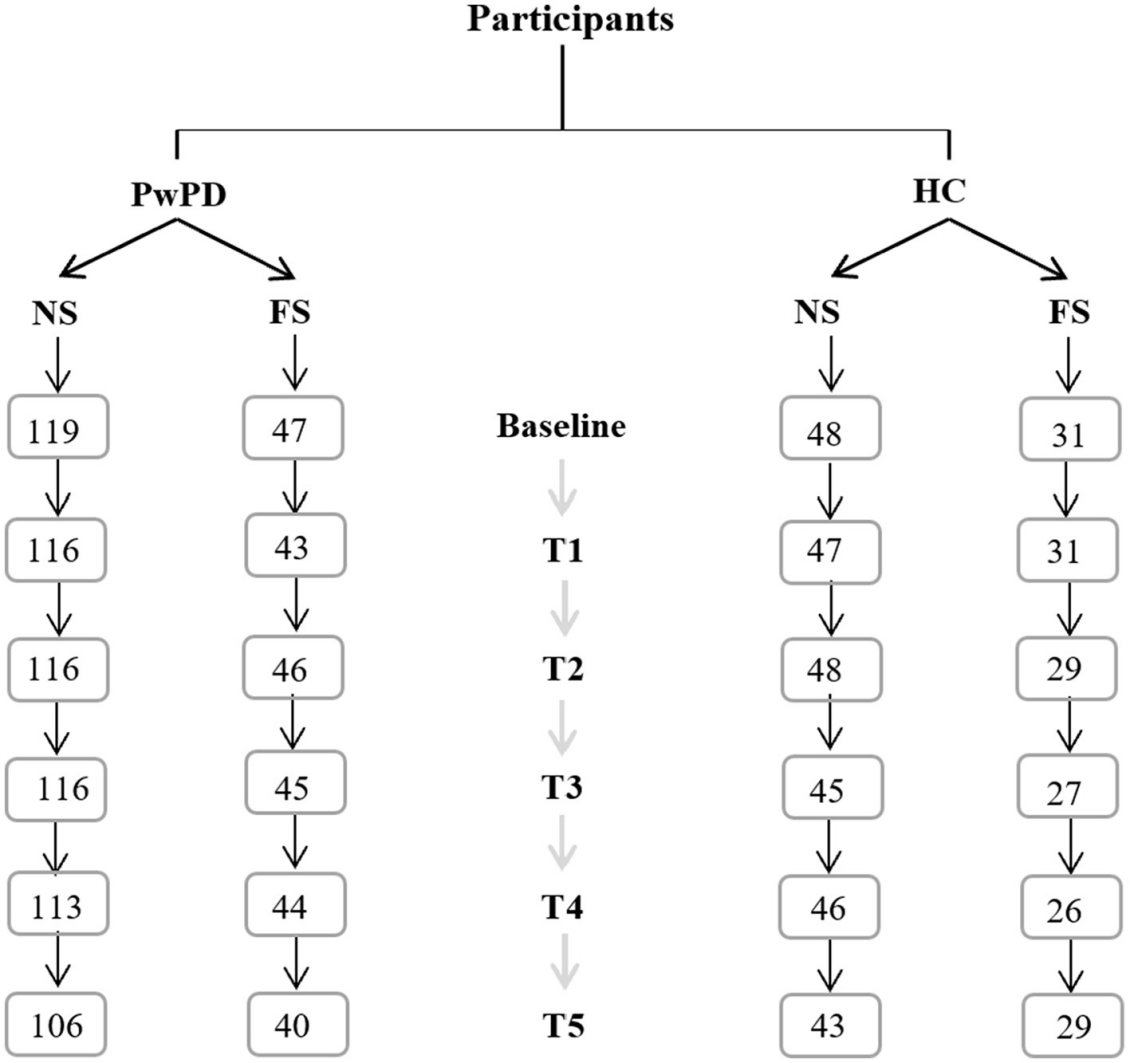
Flowchart representing the participants' samples. Participants have been divided into PwPD and healthy controls (HC). Both groups were split into participants with a regular smoking history (FS) and without a regular smoking history (NS). Excluding participants who changed diagnosis and those with a high percentage of missing data, the flowchart above shows the number of participants in each sample at each follow-up time point from baseline to the fifth year.

Participants were selected based on the availability of smoking-related data. Not all individuals from the PPMI database participated in the FOUND project. In total, 683 participants completed the lifestyle questionnaire. Among these, individuals with an excessively high proportion of missing data (30%) and those who preferred not to respond to the smoking-related questions were excluded from the analysis. Consequently, the final sample was a convenience sample consisting of all available patients who met the required reported characteristics. Data from 166 PwPD (119 Non-Smokers -NS- and 47 Former Smokers - FC) and 79 HC (48 Non-Smokers -NS- and 31 Former Smokers -FS) were analyzed. Six timepoints were considered, from T0 being the baseline and T5 being a 5-year follow-up. Attrition rates at every follow-up are reported in Figure 1.

### 2.2 Selected measures

Personal information about sex, age, education, and pharmacological therapy for PD symptoms was selected from the PPMI database (Marek et al., 2018, 2011). In addition, data from PwPD and HC included:

- *The Movement Disorder Society – Unified Parkinson's Disease Rating Scale* (UPDRS; Goetz et al., 2008; Hoehn and Yahr, 1967) Part III (ON and OFF scores) was used as an index of motor function and PD motor symptom severity.
- *The Montreal Cognitive Assessment* (MoCA; Nasreddine et al., 2012) served as an index of general cognitive status.
- The variable *cognitive state*, which classifies participants as having Normal Cognition, Mild Cognitive Impairment (MCI), or Dementia, is based on clinical evaluations of key cognitive domains, including executive functions, attention, memory, orientation, and language.
- *The Geriatric Depression Scale* (GDS; Yesavage and Sheikh, 1986) is a measure of depressive symptoms.
- The State-Trait *Anxiety Inventory* (STAI; Spielberger, 1983) is a measure of anxiety symptoms.
- Behavioral impulsivity was assessed using the *Questionnaire for Impulsive-Compulsive Disorders in PD* (QUIP; Weintraub et al., 2012).

For cigarette smoking, data were extracted from the FOUND database. Participants answered several questions about their smoking habits. Both PwPD and HC were classified as Former Smokers (FS) if they responded positively to the question: *In your lifetime, have you ever regularly smoked cigarettes, that is, at least one cigarette per day for 6 months or longer?* They were classified as Non-Smokers (NS) if they answered negatively. Only 8 individuals were current smokers; therefore, we included only former smokers (*N* = 81). Data on quantitative smoking measures (including smoking duration, age at cessation, and pack-years) were excluded from the analyses due to a high proportion of missing responses, as many participants did not provide this information when completing the questionnaire. Finally, from the FOUND database, we also considered the presence of a regular history of alcohol drinking (*In your lifetime, have you ever regularly drunk alcohol, that is, at least one drink per week for 6 months or longer?)*, as alcohol consumption and cigarette smoking are often associated (Beard et al., 2017; Jiang et al., 2014).

### 2.3 Statistical analyses

Exploratory data analyses revealed a high percentage of missing data for some variables. This was especially true for UPDRS-III (14%) and the variable cognitive state (18%). The missing data were handled using *multiple imputation by chained equations (MICE)* as implemented in the mice R package (van Buuren and Groothuis-Oudshoorn, 2011). This iterative method imputes each incomplete variable conditionally on the others through a series of regression models, repeating the cycle until convergence. The final estimates are then pooled across multiple imputed datasets to account for uncertainty due to missingness.

In this first set of analyses, we compared Former Smoker PwPD (FS-PwPD) with Non-Smoker PwPD (NS-PwPD) at each follow-up time point. We investigated between-group differences in each of the selected measures. Pearson's Chi-squared test was used for categorical variables, the Wilcoxon rank sum test for ordinal variables, and Fisher's exact test for dichotomous variables. The same analysis was performed for the HC group.

To assess the predictive effect of cigarette smoke on the progression of selected measures, we implemented multivariable multilevel regression models. We tested the impact of a regular smoking history, correcting each model for the variables of age, gender, education, time point, and the regular alcohol drinking history. Each model used one of the selected measures to assess symptomatology—UPDRS-III, MoCA, Cognitive State, GDS, STAI, QUIP—as the dependent variable. The models considered both baseline individual variation (intercept) and possible variation in individual parameters over time. Both were regarded as random effects. The same analyses were conducted for the HC group.

This analysis used data openly available from PPMI (Tier 1 Data).

## 3 Results

### 3.1 PwPD: former smokers vs. non-smokers

A total of 166 PwPD were selected at baseline, of whom 119 were NS-PwPD (72 male; 47 female; age_median_ = 59; years of education_median_ = 16) and 47 were FS-PwPD (38 male; 9 female; age_median_ = 64; years of education_median_ = 16), according to the answers to the self-completed questionnaire mentioned above. The following paragraphs will report the results divided into domains, as summarized in Table 1: Motor symptoms, Cognition, ICBs and alcohol consumption, and Depression and anxiety symptoms.

**Table 1:** Descriptive analyses for PwPD at baseline.

**Baseline**			
	**Non-smokers (*****N*** = **119)**^a^	**Former smokers (*****N*** = **47)**^a^	***p*** **-value** ^b^
15.6-2.2,-1.3242pt Age	59 (53, 65)	64 (58, 69)	0.01^*^
**Sex**			0.012^*^
Male	72 (61%)	38 (81%)	
Female	47 (39%)	9 (19%)	
Years of education	16 (15, 18)	16 (15, 18)	0.4
UPDRS-III	19 (14, 25)	18 (15, 23)	0.9
UPDRS-III (ON)	19 (14, 25)	18 (15, 23)	0.9
Medications^d^	0 (0%)	0 (0%)	
15.6-2.2,-1.3242pt GDS	1 (0, 3)	2 (1, 3)	0.2
**GDS Categorial**			0.2
< 5 (not depressed)	106 (89%)	45 (96%)	
>5 (depressed)	13 (11%)	2 (4.3%)	
QUIP_any			0.020^*^
Absent	104 (87%)	34 (72%)	
Present	15 (13%)	13 (28%)	
State-STAI	29 (23, 36)	27 (22, 36)	0.4
Trait-STAI	28 (25, 35)	27 (24, 33)	0.3
STAI	58 (49, 70)	55 (48, 67)	0.3
15.6-2.2,-1.3242pt MoCA	28 (26, 29)	27 (26, 29)	0.3
**Cognitive State**			0.5
Normal	29 (91%)	13 (100%)	
MCI	3 (9.4%)	0 (0%)	
Dementia	0 (0%)	0 (0%)	
NA^c^	87	34	
**Alcohol regular**			0.006^**^
**history**			
No	50 (42%)	9 (19%)	
Yes	69 (58%)	38 (81%)	

^a^*n* (%); Median (IQR).

^b^Fisher's exact test; Wilcoxon rank sum test; Pearson's Chi-squared test.

^c^NA: number of missing data.

^d^Use of PD Medications at the time of the Study Visit: 0 = Unmedicated for PD;1 = Levodopa; 2 = Dopamine Agonist; 3 = Others; 4 = Levodopa + Others; 5 = Levodopa + Dopamine Agonist; 6 = Dopamine Agonist + Others; 7 = Levodopa + Dopamine Agonist + Others.

^*^p < 0.05.

^**^p < 0.01.

^***^p < 0.001.

#### 3.1.1 Motor symptoms

At baseline, the UPDRS-III scores indicated mild motor symptoms in both NS-PwPD (UPDRS-IIImedian = 19) and FS-PwPD (UPDRS-IIImedian = 18), with no significant differences between the two groups (lowest *p* = 0.9).

#### 3.1.2 Cognition

At baseline, there were no significant differences in mean MoCA scores between the two groups (NS-PwPD MoCA_median_ = 28; FS-PwPD MoCA_median_ = 27; p =.3). However, the variable representing cognitive state, which divides the sample into Normal Cognition, MCI, and Dementia, showed a different distribution for PwPD with and without a smoking history. Specifically, at the third time point, the percentage of people with MCI was significantly higher in FS-PwPD (27%) than in NS-PwPD (8.6%; χ2 *Pearson* (1) = 8.95, p < 0.001). At the fourth time point, the percentage of people with MCI remained higher in FS-PwPD (20%) compared to NS-PwPD (7.6%), but this difference was no longer statistically significant (χ2 *Pearson* (2) = 4.63, *p* = 0.10); see Supplementary materials.

#### 3.1.3 ICBs and alcohol consumption

Results showed a significant difference between FS-PwPD and NS-PwPD in the QUIP scores at baseline. 28% of FS-PwPD and 13% of NS-PwPD exhibited at least one ICB (QUIP any) (OR = 2.65, 95% CI 1.15–6.13, *p* = 0.02). This difference in QUIP score between the two groups of PwPD decreased during follow-up and lost statistical significance at the fifth year (χ2 *Pearson* (1) = 2.62; *p* = 0.11). Regarding alcohol consumption, a greater proportion of FS-PwPD (79%) reported drinking alcohol regularly compared to NS-PwPD (58%, *p* = 0.013); see Supplementary materials.

#### 3.1.4 Depression and anxiety symptoms

At baseline, 11% of NS-PwPD and 4.3% of FS-PwPD presented significant depressive symptomatology according to the GDS score. Both groups experienced a mild level of anxiety (NS-PwPD state-STAI_median_ = 29; FS-PwPD state-STAI_median_ = 27), with no significant differences between groups.

### 3.2 HC: former smokers vs. non-smokers

Of the 79 healthy participants at baseline, 48 were non-smokers (NS-HC; 34 male, 14 female; median age 59; median education 18 years) and 31 were former smokers (FS-HC; 21 male, 10 female; age_median_ = 61; years of education_median_ 16).

Regarding demographics, the results indicated that NS-HC participants had, on average, more years of education (median = 18) than FS-HC participants (median = 16; *p* = 0.062). Data regarding baseline characteristics are summarized in Table 2.

**Table 2:** Descriptive statistics for healthy controls at baseline.

**Baseline**			
	**Non-smokers (*****N*** = **48)**^a^	**Former smokers (*****N*** = **31)**^a^	***p*** **-value** ^b^
15.6-2.2,-1.3242pt Age	59 (56, 69)	61 (56, 68)	0.9
**Sex**			0.8
Male	34 (71%)	21 (68%)	
Female	14 (29%)	10 (32%)	
Years of education	18 (16, 19)	16 (15, 19)	0.062
UPDRS-III	0 (0, 2)	0 (0, 2)	0.7
UPDRS-III (ON)	0 (0, 2)	0 (0, 2)	0.7
15.6-2.2,-1.3242pt GDS	1 (0, 1)	1 (0, 2)	0.7
**GDS categorial**			0.9
< 5 (not depressed)	46 (96%)	30 (97%)	
15.6-2.2,-1.3242pt >5 (depressed)	2 (4.2%)	1 (3.2%)	
**QUIP_any**			0.11
Absent	40 (83%)	21 (68%)	
Present	8 (17%)	10 (32%)	
State-STAI	23 (21, 30)	23 (20, 32)	0.9
Trait-STAI	26 (23, 31)	26 (23, 32)	0.9
STAI	51 (44, 61)	51 (44, 65)	0.8
15.6-2.2,-1.3242pt MoCA	28 (27, 29)	28 (27, 29)	0.5
**Cognitive state**			0.9
Normal	14 (100%)	3 (100%)	
MCI	0 (0%)	0 (0%)	
Dementia	34	28	
**Alcohol regular**			0.026^*^
**history**			
No	15 (31%)	3 (9.7%)	
Yes	33 (69%)	28 (90%)	

^a^*n* (%); Median (IQR).

^b^Fisher's exact test; Wilcoxon rank sum test; Pearson's Chi-squared test.

^c^NA: number of missing data.

^*^p < 0.05.

^**^p < 0.01.

^***^p < 0.001.

#### 3.2.1 Motor function

No significant differences in motor function emerged when comparing the two groups (NS-HC UPDRS-III_median_ = 0; FS-HC UPDRS-III_median_ = 0), (lowest *p* = 0.7).

#### 3.2.2 Cognition

No significant differences emerged when comparing the cognitive performance of the two groups at baseline (NS-HC MoCA_median_ = 28; FS-HC MoCA_median_ = 28) and in the other timepoints (lowest *p* = 0.14).

#### 3.2.3 ICBs and alcohol consumption

At baseline, 32% of FS-HC and 17% of NS-HC exhibited at least one Impulsive Compulsive Disorder symptom (QUIP any), though this difference was not significant (OR = 2.38, 95% CI 0.82–6.94, *p* = 0.11). Regular alcohol consumption was significantly higher among FS-HC (90%) than NS-HC (69%; *p* = 0.026).

#### 3.2.4 Depressive and anxiety symptoms

For affective symptoms, a low percentage of NS-HC (4.2%) and FS-HC (3.2%) showed significant depressive symptoms according to the GDS score, with no significant difference between the two groups at baseline. Both groups had low anxiety levels (state-STAI_median_ = 23), with no significant differences between non-smokers and former smokers at any time point (lowest *p* = 0.4); see Supplementary materials.

### 3.3 Predictive effect of cigarette smoking on PD symptom progression

Results of the regression model indicated no significant effect of cigarette smoking on the considered outcome measures in the PwPD group (lowest *p* = 0.2, β = −0.49, CI −1.2, 0.20). No significant results emerged when considering HC (lowest *p* = 0.2, log(OR) = 1.8, CI −1.1, 4.8); see Supplementary materials.

## 4 Discussion

This study aimed to clarify the relationship between cigarette smoking and the motor, cognitive, affective, and behavioral features of PD. Using the PPMI cohort, we tracked symptoms progression over 5 years. The cohort included 166 individuals with newly diagnosed de novo PD and 79 HC. At baseline, we compared former smokers and non-smokers within each group. We hypothesized that former smokers would have milder motor symptoms at diagnosis. We also anticipated that former smokers would develop ICBs and affective symptoms more rapidly, and experience faster cognitive decline, compared to non-smokers.

Contrary to our initial motor symptom hypothesis, there were no significant baseline differences between former smokers and non-smokers with PD. However, in line with our hypothesis, former smokers showed poorer cognitive performance and a notably higher prevalence of ICBs. It is worth noting that former smokers were, on average, 5 years older. This makes direct interpretations of smoking's role in cognitive differences challenging. Additionally, regression analyses revealed no significant effect of smoking history on longitudinal cognitive decline. This tempers conclusions about smoking's contribution to progressive cognitive impairment.

Furthermore, it is important to note that factors other than smoking may influence the age differences observed between the two groups. For instance, older adults tend to experience loneliness more frequently and with greater related health consequences than younger adults (Tragantzopoulou and Giannouli, 2021). This is a relevant consideration, as loneliness has been linked to a higher likelihood of smoking (Lauder et al., 2006). However, the prevalence of loneliness among older adults may sometimes be overestimated, particularly among the younger-old population (65-75) (Dykstra, 2009). In the present study, the median age of the FS-PwPD group was 64 years. Furthermore, baseline differences between FS and NS were significant among PwPD but not among healthy individuals. Nonetheless, future research should include assessments of loneliness and social isolation to understand their potential influence.

The most notable finding concerns impulsive-compulsive behaviors. De novo PwPD with a smoking history had a greater baseline presence of ICBs than non-smokers. This difference was unlikely explained by age, since ICBs are usually more common in younger PwPD (Weintraub et al., 2010). The association may reflect a direct relationship between smoking and ICBs. It is also possible that higher alcohol consumption among former smokers influenced the result. Importantly, no smoking-related differences in ICBs were found among healthy controls. This highlights a potential interaction between PD-specific neurobiology and smoking history in driving these behaviors. However, other relevant factors, such as apathy, anxiety and depression, which can affect cognitive abilities and motivational drive, may also influence the occurrence of ICDs in PwPD (Morris et al., 2025; Di Rosa et al., 2022; Giannouli and Tsolaki, 2021; Muhammed et al., 2016; Drew et al., 2020). As these domains were not considered in the present study, their potential contribution cannot be ruled out. Given that mood, anxiety and motivational symptoms are closely linked to dopaminergic and frontostriatal dysfunction (Muhammed et al., 2016; Drew et al., 2020), incorporating standardized measures of apathy, anxiety and depression in future longitudinal designs will be essential to clarify their interaction with smoking on ICD development.

Overall, these findings align with prior research linking smoking history to altered reward circuit function and increased ICB risk in PD (Carbone and Djamshidian, 2024; Valença et al., 2013). However, they contrast with meta-analytic evidence showing no significant association between smoking and ICBs (Molde et al., 2018). Our focus on a well-characterized de novo PD cohort helps address key limitations of past studies. Previous work often included medicated PwPD, making it harder to clarify smoking's role before dopaminergic treatment confounds the picture.

Smoking was linked to ICB presence at baseline, but it did not predict ICB progression after DRT initiation (Zhu et al., 2023). This highlights the crucial influence of medication status in evaluating environmental risk factors. DRT profoundly alters dopaminergic neurotransmission and ICB susceptibility. It may mask or override any prior nicotine-induced sensitization, indicating a temporal and mechanistic boundary to smoking's effects (Mansvelder and McGehee, 2002; Robinson and Berridge, 2008).

We found that former smokers with de novo PD exhibited more impulsive-compulsive behaviors at baseline. However, there was no longitudinal predictive effect of smoking on ICB progression following medication. This observation can be further contextualized through genetic and preclinical research on nicotine's neuromodulatory actions (Omaye, 2002; Quik et al., 2008; Rose et al., 2024; Ruan et al., 2023).

Genetic variability in nicotinic acetylcholine receptor subunits and dopaminergic pathway genes likely modulates individual susceptibility to smoking-induced neuroplastic changes (Keller et al., 2012; Nalls et al., 2019; Wirdefeldt et al., 2011). These genetic differences may explain why smoking history correlates with early ICB manifestation before DRT initiation.

Preclinical rodent models have demonstrated that chronic nicotine exposure leads to lasting alterations in striatal dopamine signaling and receptor dynamics. This exposure effectively “primes” the reward circuitry, increasing baseline impulsivity and compulsive tendencies (Mansvelder and McGehee, 2002; Robinson and Berridge, 2008).

The absence of a significant smoking effect on ICB progression after DRT matches evidence that dopaminergic therapy reshapes dopaminergic transmission. This therapy may override or mask prior nicotine-induced sensitization (Zhu et al., 2023). This pattern suggests a temporal and mechanistic boundary. Smoking's impact is most evident before pharmacological modulation, supporting the concept of a priming effect rather than a sustained risk driver (Noyce et al., 2012).

In healthy controls, there were no smoking-related ICB differences. This reinforces the need for PD-related neurobiological vulnerabilities, such as dopaminergic deficits and genetic predispositions, to manifest these behavioral effects (Carbone and Djamshidian, 2024; Poewe et al., 2017; Valença et al., 2013). This finding supports gene-environment interaction models. In these models, smoking-related neuroadaptations lead to pathological symptoms only in a susceptible PD context (Keller et al., 2012; Wirdefeldt et al., 2011).

These insights together suggest that cigarette smoking may contribute to early non-motor PD symptoms, particularly ICBs. The effect may occur through neuroplastic changes in reward circuits shaped by genetic background, as well as by influencing dopamine sensitivity and dopamine reserve. However, once dopaminergic therapy begins, these effects become harder to detect. This emphasizes the critical importance of medication timing when assessing environmental influences on PD (Chen et al., 2021; Mappin-Kasirer et al., 2020).

### 4.1 Limitations and future directions

Our results should be interpreted in light of the following limitations. First, the limited number of current smokers prevented us from comparing former smokers, current smokers, and non-smokers. Second, the FOUND database contained substantial missing data, requiring us to exclude several crucial variables, such as the quantity of cigarettes smoked and the duration of smoking history. Given evidence of a dose-dependent effect, this aspect could not be investigated, so our results must be interpreted with caution. Moreover, the available questionnaires lacked key indicators of reliability, such as Cronbach's alpha. Third, the age difference between former smokers and non-smokers may have affected not only cognitive performance but also other aspects of PD neuropathology and, consequently, symptomatology. Finally, important factors, like apathy and loneliness, that potentially influencing the selected outcomes, were not investigated. Future studies should, therefore, include more balanced samples of current and former smokers and obtain information about the precise dosage and duration of smoking habits, together with assessment of other crucial aspects such as apathy and loneliness.

## 5 Conclusion

In sum, the novelty of our findings lies in showing that smoking-related influences on impulsive-compulsive behaviors in PD are most evident at the de novo stage, before any dopaminergic treatment. This temporal specificity clarifies previous conflicting findings. It highlights the importance of focusing on medication-naïve PwPD to distinguish between environmental factors and pharmacological effects. Our work advances the understanding of how smoking history interacts with PD pathophysiology. It also underscores the need to consider medication status when evaluating nonmotor symptom risks in PD.

## Data Availability

Publicly available datasets were analyzed in this study. Data used in the preparation of this article were obtained from the Parkinson Progressive Marker Initiative (PPMI) database (Marek et al., 2011, 2018). For up-to-date information on the study, visit: https://www.ppmi-info.org.
