# Supplementary material for: From cigarettes to compulsions: a longitudinal study in de novo Parkinson's disease
Source: Front Psychol. 2025 Dec 17;16:1708535. doi: 10.3389/fpsyg.2025.1708535 (PMC12754602; doi:10.3389/fpsyg.2025.1708535)
Supplement: Supplementary file 1 [file Table_1.docx]

Supplementary Material

Table 1: *Descriptive analyses of PwPD at first year of follow-up.*

| **T1** | | | |
| --- | --- | --- | --- |
|  | **Non-Smokers**  **(N = 116)^1^** | **Former Smokers**  **(N = 43)^1^** | **p-value**^2^ |
| Age | 59 (53, 65) | 64 (57, 69) | 0.040* |
| Sex |  |  | 0.027* |
| Male | 70 (60%) | 34 (79%) |  |
| Female | 46 (40%) | 9 (21%) |  |
| Years of education | 16 (15, 18) | 16 (15, 18) | 0.5 |
| UPDRS-III | 24 (18, 31) | 23 (19, 27) | 0.6 |
| NA^3^ | 11 | 2 |  |
| UPDRS-III (ON) | 21 (16, 29) | 21 (16, 27) | 0.5 |
| NA^3^ | 3 | 2 |  |
| Medications^4^ |  |  | 0.4 |
| 0 | 56 (48%) | 17 (40%) |  |
| 1 | 17 (15%) | 12 (28%) |  |
| 2 | 11 (9.5%) | 3 (7.0%) |  |
| 3 | 23 (20%) | 6 (14%) |  |
| 4 | 2 (1.7%) | 2 (4.7%) |  |
| 5 | 3 (2.6%) | 1 (2.3%) |  |
| 6 | 4 (3.4%) | 2 (4.7%) |  |
| GDS | 2 (0, 3) | 1 (1, 3) | 0.9 |
| GDS Categorial |  |  | 0.2 |
| <5 (not depressed) | 101 (87%) | 41 (95%) |  |
| >5 (depressed) | 15 (13%) | 2 (4.7%) |  |
| QUIP_any |  |  | 0.8 |
| Absent | 102 (88%) | 37 (86%) |  |
| Present | 14 (12%) | 6 (14%) |  |
| State-STAI | 29 (23, 36) | 28 (25, 31) | 0.4 |
| Trait-STAI | 29 (25, 36) | 28 (26, 33) | 0.6 |
| STAI | 58 (48, 72) | 55 (50, 65) | 0.4 |
| MoCA | 28 (26, 29) | 26 (25, 29) | 0.022* |
| Cognitive State |  |  | 0.7 |
| Normal | 75 (89%) | 26 (87%) |  |
| MCI | 9 (11%) | 4 (13%) |  |
| Dementia | 0 (0%) | 0 (0%) |  |
| NA^3^ | 32 | 13 |  |
| Alcohol regular history |  |  | 0.013* |
| No | 49 (42%) | 9 (21%) |  |
| Yes | 67 (58%) | 34 (79%) |  |

*^1^n (%); Median (IQR)*

*^2^Fisher’s exact test; Wilcoxon rank sum test; Pearson’s Chi-squared test*

*^3^NA: number of missing data*

*^4^Use of PD Medications at the time of the Study Visit: 0 = Unmedicated for PD;1 = Levodopa; 2 = Dopamine Agonist; 3 = Others; 4 = Levodopa + Others; 5= Levodopa + Dopamine Agonist; 6 = Dopamine Agonist + Others; 7 = Levodopa + Dopamine Agonist + Others.*

**p < .05, ** p < .01, *** p < .001*

Table 2: *Descriptive analyses of PwPD at second year of follow-up.*

| **T2** | | | |
| --- | --- | --- | --- |
|  | **Non-Smokers**  **(N = 116)^1^** | **Former Smokers**  **(N = 46)^1^** | **p-value^2^** |
| Age | 59 (53, 65) | 64 (58, 69) | 0.011* |
| Sex |  |  | 0.015* |
| Male | 70 (60%) | 37 (80%) |  |
| Female | 46 (40%) | 9 (20%) |  |
| Years of education | 16 (16, 18) | 16 (14, 18) | 0.4 |
| NA^3^ | 25 | 8 |  |
| UPDRS-III (ON) | 21 (15, 32) | 23 (15, 30) | 0.8 |
| NA^3^ | 5 | 2 |  |
| Medications^4^ |  |  | 0.5 |
| 0 | 20 (17%) | 9 (20%) |  |
| 1 | 26 (22%) | 14 (30%) |  |
| 2 | 21 (18%) | 4 (8.7%) |  |
| 3 | 23 (20%) | 8 (17%) |  |
| 4 | 5 (4.3%) | 5 (11%) |  |
| 5 | 7 (6.0%) | 3 (6.5%) |  |
| 6 | 13 (11%) | 3 (6.5%) |  |
| 7 | 1 (0.9%) | 0 (0%) |  |
| GDS | 2 (0, 3) | 1 (0, 3) | 0.7 |
| GDS Categorial |  |  | 0.6 |
| <5 (not depressed) | 104 (90%) | 40 (87%) |  |
| >5 (depressed) | 12 (10%) | 6 (13%) |  |
| QUIP_any |  |  | 0.2 |
| Absent | 95 (82%) | 33 (72%) |  |
| Present | 21 (18%) | 13 (28%) |  |
| State-STAI | 28 (24, 34) | 31 (22, 34) | 0.7 |
| Trait-STAI | 29 (24, 35) | 31 (24, 33) | 0.8 |
| STAI | 57 (47, 71) | 61 (46, 67) | 0.7 |
| MoCA | 28 (26, 29) | 27 (24, 28) | 0.10 |
| Cognitive State |  |  | 0.8 |
| Normal | 106 (92%) | 41 (91%) |  |
| MCI | 9 (7.8%) | 4 (8.9%) |  |
| Dementia | 0 (0%) | 0 (0%) |  |
| NA^3^ | 1 | 1 |  |
| Alcohol regular history |  |  | 0.003** |
| No | 49 (42%) | 8 (17%) |  |
| Yes | 67 (58%) | 38 (83%) |  |

*^1^n (%); Median (IQR)*

*^2^Fisher’s exact test; Wilcoxon rank sum test; Pearson’s Chi-squared test*

*^3^NA: number of missing data*

*^4^Use of PD Medications at the time of the Study Visit: 0 = Unmedicated for PD;1 = Levodopa; 2 = Dopamine Agonist; 3 = Others; 4 = Levodopa + Others; 5= Levodopa + Dopamine Agonist; 6 = Dopamine Agonist + Others; 7 = Levodopa + Dopamine Agonist + Others.*

**p < .05, ** p < .01, *** p < .001*

Table 3: *Descriptive analyses of patients with PD diagnosis at third year of follow-up.*

| **T3** | | | |
| --- | --- | --- | --- |
|  | **Non-Smokers**  **(N = 116)^1^** | **Former Smokers**  **(N = 45)^1^** | **p-value**^2^ |
| Age | 58 (52, 65) | 64 (57, 69) | 0.014* |
| Sex |  |  | 0.018* |
| Male | 70 (60%) | 36 (80%) |  |
| Female | 46 (40%) | 9 (20%) |  |
| Years of education | 16 (15, 18) | 16 (14, 18) | 0.3 |
| UPDRS-III | 28 (22, 36) | 31 (24, 39) | 0.3 |
| NA^3^ | 28 | 6 |  |
| UPDRS-III (ON) | 22 (13, 32) | 27 (17, 34) | 0.11 |
| NA^3^ | 7 | 2 |  |
| Medications^4^ |  |  | 0.6 |
| 0 | 8 (6.9%) | 4 (8.9%) |  |
| 1 | 32 (28%) | 15 (33%) |  |
| 2 | 20 (17%) | 3 (6.7%) |  |
| 3 | 12 (10%) | 7 (16%) |  |
| 4 | 13 (11%) | 3 (6.7%) |  |
| 5 | 17 (15%) | 7 (16%) |  |
| 6 | 6 (5.2%) | 4 (8.9%) |  |
| 7 | 8 (6.9%) | 2 (4.4%) |  |
| GDS | 2 (1, 3) | 2 (1, 3) | 0.5 |
| GDS Categorial |  |  | 0.9 |
| <5 (not depressed) | 103 (89%) | 40 (89%) |  |
| >5 (depressed) | 13 (11%) | 5 (11%) |  |
| QUIP_any |  |  | 0.3 |
| Absent | 91 (78%) | 32 (71%) |  |
| Present | 25 (22%) | 13 (29%) |  |
| State-STAI | 28 (22, 36) | 28 (24, 34) | 0.9 |
| NA^3^ | 0 | 1 |  |
| Trait-STAI | 29 (25, 35) | 29 (24, 33) | 0.9 |
| NA^3^ | 0 | 1 |  |
| STAI | 56 (49, 71) | 58 (47, 69) | 0.9 |
| NA^3^ | 0 | 1 |  |
| MoCA | 28 (26, 29) | 27 (25, 29) | 0.015* |
| NA^3^ | 0 | 1 |  |
| Cognitive State |  |  | 0.005** |
| Normal | 106 (91%) | 33 (73%) |  |
| MCI | 10 (8.6%) | 12 (27%) |  |
| Dementia | 0 (0%) | 0 (0%) |  |
| Alcohol regular history |  |  | 0.014* |
| No | 47 (41%) | 9 (20%) |  |
| Yes | 69 (59%) | 36 (80%) |  |

*^1^n (%); Median (IQR)*

*^2^Fisher’s exact test; Wilcoxon rank sum test; Pearson’s Chi-squared test*

*^3^NA: number of missing data*

*^4^Use of PD Medications at the time of the Study Visit: 0 = Unmedicated for PD;1 = Levodopa; 2 = Dopamine Agonist; 3 = Others; 4 = Levodopa + Others; 5= Levodopa + Dopamine Agonist; 6 = Dopamine Agonist + Others; 7 = Levodopa + Dopamine Agonist + Others.*

**p < .05, ** p < .01, *** p < .001*

Table 4: *Descriptive analyses of PwPD at fourth year of follow-up.*

| **T4** | | | |
| --- | --- | --- | --- |
|  | **Non-Smokers**  **(N = 113)^1^** | **Former Smokers**  **(N = 44)^1^** | **p-value**^2^ |
| Age | 58 (52, 64) | 64 (58, 69) | 0.005** |
| Sex |  |  | 0.022* |
| Male | 68 (60%) | 35 (80%) |  |
| Female | 45 (40%) | 9 (20%) |  |
| Years of education | 16 (16, 18) | 16 (15, 18) | 0.3 |
| UPDRS-III | 29 (22, 38) | 33 (27, 39) | 0.2 |
| NA^3^ | 20 | 6 |  |
| UPDRS-III (ON) | 20 (12, 31) | 25 (16, 34) | 0.13 |
| NA^3^ | 5 | 1 |  |
| Medications^4^ |  |  |  |
| 0 | 7 (6.2%) | 1 (2.3%) |  |
| 1 | 31 (27%) | 20 (47%) |  |
| 2 | 9 (8.0%) | 0 (0%) |  |
| 3 | 7 (6.2%) | 5 (12%) |  |
| 4 | 16 (14%) | 4 (9.3%) |  |
| 5 | 28 (25%) | 4 (9.3%) |  |
| 6 | 6 (5.3%) | 6 (14%) |  |
| 7 | 9 (8.0%) | 3 (7.0%) |  |
| NA^3^ | 0 | 1 |  |
| GDS | 1 (0, 3) | 2 (1, 3) | 0.4 |
| NA^3^ | 0 | 1 |  |
| GDS Cagorial |  |  | 0.5 |
| <5 (not depressed) | 98 (87%) | 39 (91%) |  |
| >5 (depressed) | 15 (13%) | 4 (9.3%) |  |
| NA^3^ | 0 | 1 |  |
| QUIP_any |  |  | 0.9 |
| Absent | 86 (76%) | 33 (77%) |  |
| Present | 27 (24%) | 10 (23%) |  |
| NA^3^ | 0 | 1 |  |
| State-STAI | 29 (23, 37) | 28 (22, 34) | 0.3 |
| NA | 0 | 1 |  |
| Trait-STAI | 30 (26, 38) | 27 (24, 35) | 0.058 |
| NA^3^ | 0 | 1 |  |
| STAI | 60 (48, 73) | 52 (46, 69) | 0.11 |
| NA^3^ | 0 | 1 |  |
| MoCA | 28 (26, 29) | 28 (26, 29) | 0.2 |
| NA^3^ | 0 | 2 |  |
| Cognitive State |  |  | 0.8 |
| Normal | 99 (88%) | 38 (90%) |  |
| MCI | 14 (12%) | 4 (9.5%) |  |
| Dementia | 0 (0%) | 0 (0%) |  |
| NA^3^ | 0 | 2 |  |
| Alcohol regular history |  |  | 0.006** |
| No | 47 (42%) | 8 (18%) |  |
| Yes | 66 (58%) | 36 (82%) |  |

*^1^n (%); Median (IQR)*

*^2^Fisher’s exact test; Wilcoxon rank sum test; Pearson’s Chi-squared test*

*^3^NA: number of missing data*

*^4^Use of PD Medications at the time of the Study Visit: 0 = Unmedicated for PD;1 = Levodopa; 2 = Dopamine Agonist; 3 = Others; 4 = Levodopa + Others; 5= Levodopa + Dopamine Agonist; 6 = Dopamine Agonist + Others; 7 = Levodopa + Dopamine Agonist + Others.*

**p < .05, ** p < .01, *** p < .001*

Table 5: *Descriptive analyses of PwPD at fifth year of follow-up.*

| **T5** | | | |
| --- | --- | --- | --- |
|  | **Non-Smokers**  **(N = 106)^1^** | **Former Smokers**  **(N = 40)^1^** | **p-value**^2^ |
| Age | 58 (52, 66) | 64 (58, 69) | 0.010* |
| Sex |  |  | 0.033* |
| Male | 62 (58%) | 31 (78%) |  |
| Female | 44 (42%) | 9 (23%) |  |
| Years of education | 16 (16, 18) | 16 (16, 18) | 0.5 |
| UPDRS-III | 30 (20, 39) | 32 (25, 39) | 0.4 |
| NA^3^ | 26 | 3 |  |
| UPDRS-III (ON) | 20 (13, 30) | 24 (15, 33) | 0.10 |
| NA^3^ | 2 | 1 |  |
| Medications^4^ |  |  | 0.3 |
| 0 | 4 (3.8%) | 1 (2.5%) |  |
| 1 | 31 (29%) | 18 (45%) |  |
| 2 | 6 (5.7%) | 1 (2.5%) |  |
| 3 | 2 (1.9%) | 2 (5.0%) |  |
| 4 | 22 (21%) | 7 (18%) |  |
| 5 | 23 (22%) | 4 (10%) |  |
| 6 | 5 (4.7%) | 4 (10%) |  |
| 7 | 13 (12%) | 3 (7.5%) |  |
| GDS | 1 (1, 3) | 2 (1, 3) | 0.2 |
| GDS Cagorial |  |  | 0.9 |
| <5 (not depressed) | 92 (87%) | 35 (88%) |  |
| >5 (depressed) | 14 (13%) | 5 (13%) |  |
| QUIP_any |  |  | 0.11 |
| Absent | 85 (80%) | 27 (68%) |  |
| Present | 21 (20%) | 13 (33%) |  |
| State-STAI | 27 (23, 33) | 30 (23, 35) | 0.4 |
| NA^3^ | 1 | 0 |  |
| Trait-STAI | 30 (24, 36) | 29 (23, 35) | 0.5 |
| STAI | 56 (49, 69) | 60 (46, 68) | 0.9 |
| NA^3^ | 1 | 0 |  |
| MoCA | 28 (27, 30) | 28 (26, 29) | 0.14 |
| Cognitive State |  |  | 0.087 |
| Normal | 95 (90%) | 31 (78%) |  |
| MCI | 8 (7.6%) | 8 (20%) |  |
| Dementia | 2 (1.9%) | 1 (2.5%) |  |
| NA^3^ | 1 | 0 |  |
| Alcohol regular history |  |  | 0.007** |
| No | 44 (42%) | 7 (18%) |  |
| Yes | 62 (58%) | 33 (83%) |  |

*^1^n (%); Median (IQR)*

*^2^Fisher’s exact test; Wilcoxon rank sum test; Pearson’s Chi-squared test*

*^3^NA: number of missing data*

*^4^Use of PD Medications at the time of the Study Visit: 0 = Unmedicated for PD;1 = Levodopa; 2 = Dopamine Agonist; 3 = Others; 4 = Levodopa + Others; 5= Levodopa + Dopamine Agonist; 6 = Dopamine Agonist + Others; 7 = Levodopa + Dopamine Agonist + Others.*

**p < .05, ** p < .01, *** p < .001*

Table 6: *Results of the regression model with the selected measures as dependent variable of each model.*

| **UPDRS-III** | | | |  | **UPDRS-III (ON)** | | | |
| --- | --- | --- | --- | --- | --- | --- | --- | --- |
|  | **Beta** | **95% CI^1^** | **p-value** |  |  | **Beta** | **95% CI^1^** | **p-value** |
| Age | 0.08 | -0.06, 0.22 | 0.2 |  | Age | 0.05 | -0.09, 0.19 | 0.5 |
| Sex |  |  |  |  | Sex |  |  |  |
| Male | — | — |  |  | Male | — | — |  |
| Female | -1.9 | -4.8, 1.0 | 0.2 |  | Female | -1.7 | -4.7, 1.3 | 0.3 |
| Years of education | 0.06 | -0.44, 0.56 | 0.8 |  | Years of education | -0.01 | -0.53, 0.51 | 0.9 |
| Alcohol regular history |  |  |  |  | Alcohol regular history |  |  |  |
| No | — | — |  |  | No | — | — |  |
| Yes | 1.8 | -1.0, 4.7 | 0.2 |  | Yes | 2.1 | -0.89, 5.0 | 0.2 |
| Timepoints | 0.98 | 0.82, 1.1 | <0.001*** |  | Timepoints | 0.21 | 0.04, 0.38 | 0.014* |
| Cigarettes smoke history |  |  |  |  | Cigarettes smoke history |  |  |  |
| No | — | — |  |  | No | — | — |  |
| Yes | -2.0 | -4.9, 0.84 | 0.2 |  | Yes | -1.8 | -4.8, 1.2 | 0.2 |
| *^1^CI = Confidence Interval*  **p < .05, ** p < .01, *** p < .001* | | | |  | *^1^CI = Confidence Interval*  **p < .05, ** p < .01, *** p < .001* | | | |
|  |  |  |  |  |  |  |  |  |
| **GDS** | | | |  | **STAI** | | | |
|  | **Beta** | **95% CI^1^** | **p-value** |  |  | **Beta** | **95% CI^1^** | **p-value** |
| Age | -0.04 | -0.07, -0.01 | 0.024* |  | Age | -0.33 | -0.60, -0.06 | 0.015* |
| Sex |  |  |  |  | Sex |  |  |  |
| Male | — | — |  |  | Male | — | — |  |
| Female | 0.46 | -0.26, 1.2 | 0.2 |  | Female | 2.8 | -2.9, 8.5 | 0.3 |
| Years of education | 0.02 | -0.10, 0.15 | 0.7 |  | Years of education | -0.06 | -1.0, 0.93 | 0.9 |
| Alcohol regular history |  |  |  |  | Alcohol regular history |  |  |  |
| No | — | — |  |  | No | — | — |  |
| Yes | 0.19 | -0.52, 0.90 | 0.6 |  | Yes | 1.9 | -3.7, 7.5 | 0.5 |
| Timepoints | 0.03 | 0.01, 0.06 | 0.021* |  | Timepoints | 0.00 | -0.20, 0.20 | 0.9 |
| Cigarettes smoke history |  |  |  |  | Cigarettes smoke history |  |  |  |
| No | — | — |  |  | No | — | — |  |
| Yes | -0.01 | -0.72, 0.70 | 0.9 |  | Yes | -2.1 | -7.7, 3.6 | 0.5 |
| *^1^CI = Confidence Interval*  **p < .05, ** p < .01, *** p < .001* | | | |  | *^1^CI = Confidence Interval*  **p < .05, ** p < .01, *** p < .001* | | | |
|  |  |  |  |  |  |  |  |  |
| **Trai-STAI** | | | |  | **State-STAI** | | | |
|  | **Beta** | **95% CI^1^** | **p-value** |  |  | **Beta** | **95% CI^1^** | **p-value** |
| Age | -0.23 | -0.36, -0.10 | <0.001*** |  | Age | -0.10 | -0.26, 0.05 | 0.2 |
| Sex |  |  |  |  | Sex |  |  |  |
| Male | — | — |  |  | Male | — | — |  |
| Female | 1.7 | -1.2, 4.6 | 0.2 |  | Female | 1.1 | -2.2, 4.4 | 0.5 |
| Years of education | 0.12 | -0.38, 0.61 | 0.6 |  | Years of education | -0.18 | -0.75, 0.39 | 0.5 |
| Alcohol regular history |  |  |  |  | Alcohol regular history |  |  |  |
| No | — | — |  |  | No | — | — |  |
| Yes | 0.38 | -2.4, 3.2 | 0.8 |  | Yes | 1.5 | -1.7, 4.8 | 0.3 |
| Timepoints | 0.06 | -0.04, 0.16 | 0.2 |  | Timepoints | -0.06 | -0.18, 0.06 | 0.3 |
| Cigarettes smoke history |  |  |  |  | Cigarettes smoke history |  |  |  |
| No | — | — |  |  | No | — | — |  |
| Yes | -0.41 | -3.2, 2.4 | 0.8 |  | Yes | -1.7 | -4.9, 1.6 | 0.3 |
| *^1^CI = Confidence Interval*  **p < .05, ** p < .01, *** p < .001* | | | |  | *^1^CI = Confidence Interval*  **p < .05, ** p < .01, *** p < .001* | | | |
|  |  |  |  |  |  |  |  |  |
| **MoCA** | | | |  | **Cognitive State** | | | |
|  | **Beta** | **95% CI^1^** | **p-value** |  |  | **log(OR)** | **95% CI^1^** | **p-value** |
| Age | -0.07 | -0.10, -0.03 | <0.001*** |  | Age | 0.11 | 0.02, 0.20 | 0.020 |
| Sex |  |  |  |  | Sex |  |  |  |
| Male | — | — |  |  | Male | — | — |  |
| Female | 0.94 | 0.24, 1.6 | 0.009** |  | Female | -1.3 | -3.2, 0.55 | 0.2 |
| Alcohol regular history |  |  |  |  | Years of education | 0.00 | -0.34, 0.34 | 0.9 |
| No | — | — |  |  | Alcohol regular history |  |  |  |
| Yes | 0.54 | -0.15, 1.2 | 0.12 |  | No | — | — |  |
| Timepoints | 0.01 | -0.03, 0.04 | 0.7 |  | Yes | -0.61 | -2.5, 1.2 | 0.5 |
| Cigarettes smoke history |  |  |  |  | Timepoints | -0.59 | -0.83, -0.36 | <0.001*** |
| No | — | — |  |  | Cigarettes smoke history |  |  |  |
| Yes | -0.49 | -1.2, 0.20 | 0.2 |  | No | — | — |  |
| *CI = Confidence Interval*  **p < .05, ** p < .01, *** p < .001* | | | |  | Yes | 0.43 | -1.4, 2.3 | 0.6 |
|  |  |  |  |  | *^1^OR = Odds Ratio, CI = Confidence Interval*  **p < .05, ** p < .01, *** p < .001* | | | |
|  |  |  |  |  |  |  |  |  |
| **QUIP_any** | | | |  |  | | | |
|  | **log(OR)** | **95% CI^1^** | **p-value** |  |  |  |  |  |
| Age | -0.10 | -0.27, 0.07 | 0.2 |  |  |  |  |  |
| Sex |  |  |  |  |  |  |  |  |
| Male | — | — |  |  |  |  |  |  |
| Female | -0.81 | -4.4, 2.8 | 0.7 |  |  |  |  |  |
| Years of education | -0.10 | -0.69, 0.48 | 0.7 |  |  |  |  |  |
| Alcohol regular history |  |  |  |  |  |  |  |  |
| No | — | — |  |  |  |  |  |  |
| Yes | -0.65 | -4.1, 2.8 | 0.7 |  |  |  |  |  |
| Timepoints | -0.08 | -0.37, 0.22 | 0.6 |  |  |  |  |  |
| Cigarettes smoke history |  |  |  |  |  |  |  |  |
| No | — | — |  |  |  |  |  |  |
| Yes | 1.5 | -1.8, 4.7 | 0.4 |  |  |  |  |  |
| *^1^OR = Odds Ratio, CI = Confidence Interval*  **p < .05, ** p < .01, *** p < .001* | | | |  |  | | | |

Table 7: *Descriptive analyses of healthy controls at the first year of follow-up.*

| **T1** | | | |
| --- | --- | --- | --- |
|  | **Non-Smokers**  **(N = 47)^1^** | **Former Smokers**  **(N = 31)^1^** | **p-value^2^** |
| Age | 59 (56, 69) | 61 (56, 68) | 0.9 |
| Sex |  |  | 0.8 |
| Male | 33 (70%) | 21 (68%) |  |
| Female | 14 (30%) | 10 (32%) |  |
| Years of education | 18 (16, 19) | 16 (15, 19) | 0.057 |
| UPDRS-III | 0 (0, 2) | 1 (0, 2) | 0.6 |
| UPDRS-III (ON) | 0 (0, 2) | 1 (0, 2) | 0.6 |
| GDS | 1 (0, 1) | 1 (0, 1) | 0.2 |
| GDS Categorial |  |  | 0.6 |
| <5 (not depressed) | 46 (98%) | 29 (94%) |  |
| >5 (depressed) | 1 (2.1%) | 2 (6.5%) |  |
| QUIP_any |  |  | 0.8 |
| Absent | 39 (83%) | 25 (81%) |  |
| Presnt | 8 (17%) | 6 (19%) |  |
| State-STAI | 23 (20, 28) | 23 (21, 27) | 0.8 |
| Trait-STAI | 24 (22, 31) | 26 (22, 31) | 0.8 |
| STAI | 51 (44, 57) | 48 (44, 58) | 0.9 |
| MoCA | 28 (26, 28) | 27 (26, 29) | 0.7 |
| Cognitive State |  |  | 0.4 |
| Normal | 26 (100%) | 15 (94%) |  |
| MCI | 0 (0%) | 1 (6.3%) |  |
| NA^3^ | 21 | 15 |  |
| Alcohol regular history |  |  | 0.023* |
| No | 15 (32%) | 3 (9.7%) |  |
| Yes | 32 (68%) | 28 (90%) |  |

*^1^n (%); Median (IQR)*

*^2^Fisher’s exact test; Wilcoxon rank sum test; Pearson’s Chi-squared test*

*^3^NA: number of missing data*

**p < .05, ** p < .01, *** p < .001*

Table 8: *Descriptive analyses of healthy controls at second year of follow-up.*

| **T2** | | | |
| --- | --- | --- | --- |
|  | **Non-Smokers**  **(N = 48)^1^** | **Former Smokers**  **(N = 29)^1^** | **p-value^2^** |
| Age | 59 (56, 69) | 61 (56, 68) | 0.9 |
| Sex |  |  | 0.9 |
| Male | 34 (71%) | 20 (69%) |  |
| Female | 14 (29%) | 9 (31%) |  |
| Years of education | 18 (16, 19) | 16 (15, 19) | 0.056 |
| UPDRS-III | 0 (0, 1) | 0 (0, 2) | 0.9 |
| UPDRS-III (ON) | 0 (0, 1) | 0 (0, 2) | 0.9 |
| GDS | 0 (0, 1) | 1 (0, 1) | 0.4 |
| GDS Categorial |  |  | 0.6 |
| <5 (not depressed) | 47 (98%) | 27 (93%) |  |
| >5 (depressed) | 1 (2.1%) | 2 (6.9%) |  |
| QUIP_any |  |  | 0.9 |
| Absent | 41 (85%) | 25 (86%) |  |
| Present | 7 (15%) | 4 (14%) |  |
| State-STAI | 26 (20, 29) | 23 (20, 32) | 0.9 |
| Trait-STAI | 27 (23, 31) | 26 (22, 32) | 0.7 |
| STAI | 51 (43, 61) | 49 (42, 62) | 0.8 |
| MoCA | 28 (26, 29) | 27 (25, 29) | 0.14 |
| Cognitive State |  |  | 0.9 |
| Normal | 47 (100%) | 28 (100%) |  |
| MCI | 0 (0%) | 0 (0%) |  |
| NA^3^ | 1 | 1 |  |
| Alcohol regular history |  |  | 0.004** |
| No | 15 (31%) | 1 (3.4%) |  |
| Yes | 33 (69%) | 28 (97%) |  |

*^1^n (%); Median (IQR)*

*^2^Fisher’s exact test; Wilcoxon rank sum test; Pearson’s Chi-squared test*

*^3^NA: number of missing data*

**p < .05, ** p < .01, *** p < .001*

Table 9: *Descriptive analyses of healthy controls at year of third follow-up.*

| **T3** | | | |
| --- | --- | --- | --- |
|  | **Non-Smokers**  **(N = 45)^1^** | **Former Smokers**  **(N = 27)^1^** | **p-value^2^** |
| Age | 59 (56, 69) | 61 (57, 68) | 0.7 |
| Sex |  |  | 0.7 |
| Male | 32 (71%) | 18 (67%) |  |
| Female | 13 (29%) | 9 (33%) |  |
| Years of education | 18 (16, 19) | 16 (15, 19) | 0.065 |
| UPDRS-III | 0 (0, 1) | 0 (0, 1) | 0.7 |
| UPDRS-III (ON) | 0 (0, 1) | 0 (0, 1) | 0.7 |
| GDS | 0 (0, 1) | 1 (0, 2) | 0.011* |
| GDS Categorial |  |  | 0.4 |
| <5 (not depressed) | 45 (100%) | 26 (96%) |  |
| >5 (depressed) | 0 (0%) | 1 (3.7%) |  |
| QUIP_any |  |  | 0.8 |
| Absent | 38 (84%) | 22 (81%) |  |
| Present | 7 (16%) | 5 (19%) |  |
| State-STAI | 23 (20, 27) | 23 (20, 28) | 0.7 |
| NA^3^ | 1 | 0 |  |
| Trait-STAI | 26 (22, 30) | 27 (23, 30) | 0.5 |
| STAI | 51 (44, 57) | 50 (44, 57) | 0.6 |
| NA^3^ | 1 | 0 |  |
| MoCA | 28 (26, 29) | 28 (27, 29) | 0.8 |
| Cognitive State |  |  | 0.9 |
| Normal | 45 (100%) | 26 (100%) |  |
| MCI | 0 (0%) | 0 (0%) |  |
| NA^3^ | 0 | 1 |  |
| Alcohol regular history |  |  | 0.035* |
| No | 15 (33%) | 3 (11%) |  |
| Yes | 30 (67%) | 24 (89%) |  |

*^1^n (%); Median (IQR)*

*^2^Fisher’s exact test; Wilcoxon rank sum test; Pearson’s Chi-squared test*

*^3^NA: number of missing data*

**p < .05, ** p < .01, *** p < .001*

Table 10: *Descriptive analyses of healthy controls at fourth year of follow-up.*

| **T4** | | | |
| --- | --- | --- | --- |
|  | **Non-Smokers**  **(N = 46)^1^** | **Former Smokers**  **(N = 26)^1^** | **p-value^2^** |
| Age | 59 (56, 69) | 64 (56, 68) | 0.8 |
| Sex |  |  | 0.9 |
| Male | 32 (70%) | 18 (69%) |  |
| Female | 14 (30%) | 8 (31%) |  |
| Years of education | 18 (16, 19) | 16 (14, 19) | 0.2 |
| UPDRS-III | 0 (0, 2) | 1 (0, 2) | 0.9 |
| UPDRS-III (ON) | 0 (0, 2) | 1 (0, 2) | 0.9 |
| GDS | 1 (0, 2) | 1 (0, 2) | 0.7 |
| GDS Categorial |  |  | 0.9 |
| <5 (not depressed) | 46 (100%) | 26 (100%) |  |
| >5 (depressed) | 0 (0%) | 0 (0%) |  |
| QUIP_any |  |  | 0.5 |
| Absent | 37 (80%) | 23 (88%) |  |
| Present | 9 (20%) | 3 (12%) |  |
| State-STAI | 24 (21, 28) | 23 (21, 29) | 0.6 |
| Trait-STAI | 26 (23, 31) | 28 (23, 32) | 0.4 |
| STAI | 50 (44, 55) | 51 (44, 59) | 0.5 |
| MoCA | 28 (26, 30) | 28 (27, 30) | 0.9 |
| Cognitive State |  |  | 0.4 |
| Normal | 46 (100%) | 25 (96%) |  |
| MCI | 0 (0%) | 1 (3.8%) |  |
| Alcohol regular history |  |  | 0.017* |
| No | 15 (33%) | 2 (7.7%) |  |
| Yes | 31 (67%) | 24 (92%) |  |

*^1^n (%); Median (IQR)*

*^2^Fisher’s exact test; Wilcoxon rank sum test; Pearson’s Chi-squared test*

*^3^NA: number of missing data*

**p < .05, ** p < .01, *** p < .001*

Table 11: *Descriptive analyses of healthy controls at fifth year of follow-up.*

| **T5** | | | |
| --- | --- | --- | --- |
|  | **Non-Smokers**  **(N = 43)^1^** | **Former Smokers**  **(N = 29)^1^** | **p-value^2^** |
| Age | 59 (56, 69) | 61 (56, 68) | 0.8 |
| Sex |  |  | 0.9 |
| Male | 30 (70%) | 20 (69%) |  |
| Female | 13 (30%) | 9 (31%) |  |
| Years of education | 18 (16, 20) | 16 (15, 19) | 0.12 |
| UPDRS-III | 1 (0, 3) | 1 (0, 3) | 0.5 |
| UPDRS-III (ON) | 1 (0, 3) | 1 (0, 3) | 0.5 |
| GDS | 0 (0, 1) | 1 (1, 2) | 0.001** |
| GDS Categorial |  |  | 0.9 |
| <5 (not depressed) | 43 (100%) | 29 (100%) |  |
| >5 (depressed) | 0 (0%) | 0 (0%) |  |
| QUIP_any |  |  | 0.9 |
| Absent | 36 (84%) | 25 (86%) |  |
| Present | 7 (16%) | 4 (14%) |  |
| State-STAI | 25 (21, 31) | 23 (21, 27) | 0.4 |
| Trait-STAI | 24 (21, 30) | 25 (22, 28) | 0.7 |
| STAI | 50 (43, 59) | 49 (43, 56) | 0.8 |
| MoCA | 28 (27, 30) | 28 (27, 30) | 0.7 |
| Cognitive State |  |  | 0.9 |
| Normal | 42 (98%) | 28 (97%) |  |
| MCI | 1 (2.3%) | 1 (3.4%) |  |
| Alcohol regular history |  |  | 0.029* |
| No | 14 (33%) | 3 (10%) |  |
| Yes | 29 (67%) | 26 (90%) |  |

*^1^n (%); Median (IQR)*

*^2^Fisher’s exact test; Wilcoxon rank sum test; Pearson’s Chi-squared test*

*^3^NA: number of missing data*

**p < .05, ** p < .01, *** p < .001*

Table 12: *Results of the regression model with the selected measures as dependent variable of each model.*

| **UPDRS-III** | | | |  | **UPDRS-III (ON)** | | | |
| --- | --- | --- | --- | --- | --- | --- | --- | --- |
|  | **Beta** | **95% CI^1^** | **p-value** |  |  | **Beta** | **95% CI** | **p-value** |
| Age | 0.01 | -0.02, 0.05 | 0.4 |  | Age | 0.01 | -0.02, 0.05 | 0.4 |
| Sex |  |  |  |  | Sex |  |  |  |
| Male | — | — |  |  | Male | — | — |  |
| Female | 0.05 | -0.69, 0.80 | 0.9 |  | Female | 0.05 | -0.69, 0.80 | 0.9 |
| Years of education | -0.05 | -0.19, 0.09 | 0.5 |  | Years of education | -0.05 | -0.19, 0.09 | 0.5 |
| Alcohol regular history |  |  |  |  | Alcohol regular history |  |  |  |
| No | — | — |  |  | No | — | — |  |
| Yes | 0.35 | -0.48, 1.2 | 0.4 |  | Yes | 0.35 | -0.48, 1.2 | 0.4 |
| Timepoints | 0.07 | 0.02, 0.12 | 0.004** |  | Timepoints | 0.07 | 0.02, 0.12 | 0.004** |
| Cigarettes smoke history |  |  |  |  | Cigarettes smoke history |  |  |  |
| No | — | — |  |  | No | — | — |  |
| Yes | -0.26 | -0.98, 0.46 | 0.5 |  | Yes | -0.26 | -0.98, 0.46 | 0.5 |
| *^1^CI = Confidence Interval*  **p < .05, ** p < .01, *** p < .001* | | | |  | *^1^CI = Confidence Interval*  **p < .05, ** p < .01, *** p < .001* | | | |
| **GDS** | | | |  | **STAI** | | | |
|  | **Beta** | **95% CI^1^** | **p-value** |  |  | **Beta** | **95% CI^1^** | **p-value** |
| Age | -0.01 | -0.04, 0.03 | 0.7 |  | Age | -0.15 | -0.44, 0.15 | 0.3 |
| Sex |  |  |  |  | Sex |  |  |  |
| Male | — | — |  |  | Male | — | — |  |
| Female | 0.15 | -0.55, 0.84 | 0.7 |  | Female | 0.76 | -5.5, 7.1 | 0.8 |
| Years of education | -0.07 | -0.20, 0.06 | 0.3 |  | Years of education | -1.0 | -2.2, 0.15 | 0.086 |
| Alcohol regular history |  |  |  |  | Alcohol regular history |  |  |  |
| No | — | — |  |  | No | — | — |  |
| Yes | -0.16 | -0.94, 0.62 | 0.7 |  | Yes | 2.8 | -4.2, 9.9 | 0.4 |
| Timepoints | -0.01 | -0.04, 0.02 | 0.5 |  | Timepoints | -0.14 | -0.32, 0.04 | 0.13 |
| Cigarettes smoke history |  |  |  |  | Cigarettes smoke history |  |  |  |
| No | — | — |  |  | No | — | — |  |
| Yes | 0.13 | -0.54, 0.80 | 0.7 |  | Yes | 0.41 | -5.7, 6.5 | 0.9 |
| *^1^CI = Confidence Interval*  **p < .05, ** p < .01, *** p < .001* | | | |  | *^1^CI = Confidence Interval*  **p < .05, ** p < .01, *** p < .001* | | | |
| **Trait-STAI** | | | |  | **State-STAI** | | | |
|  | **Beta** | **95% CI^1^** | **p-value** |  |  | **Beta** | **95% CI^1^** | **p-value** |
| Age | -0.06 | -0.23, 0.10 | 0.4 |  | Age | -0.08 | -0.24, 0.07 | 0.3 |
| Sex |  |  |  |  | Sex |  |  |  |
| Male | — | — |  |  | Male | — | — |  |
| Female | 1.0 | -2.5, 4.6 | 0.6 |  | Female | -0.26 | -3.6, 3.1 | 0.9 |
| Years of education | -0.58 | -1.2, 0.08 | 0.086 |  | Years of education | -0.45 | -1.1, 0.18 | 0.2 |
| Alcohol regular history |  |  |  |  | Alcohol regular history |  |  |  |
| No | — | — |  |  | No | — | — |  |
| Yes | 1.3 | -2.7, 5.3 | 0.5 |  | Yes | 1.5 | -2.2, 5.3 | 0.4 |
| Timepoints | -0.09 | -0.18, 0.01 | 0.075 |  | Timepoints | -0.05 | -0.17, 0.07 | 0.4 |
| Cigarettes smoke history |  |  |  |  | Cigarettes smoke history |  |  |  |
| No | — | — |  |  | No | — | — |  |
| Yes | 0.04 | -3.4, 3.5 | 0.9 |  | Yes | 0.38 | -2.9, 3.6 | 0.8 |
| *^1^CI = Confidence Interval*  **p < .05, ** p < .01, *** p < .001* | | | |  | *^1^CI = Confidence Interval*  **p < .05, ** p < .01, *** p < .001* | | | |
|  |  |  |  |  |  |  |  |  |
| **MoCA** | | | |  | **Cognitive State** | | | |
|  | **Beta** | **95% CI^1^** | **p-value** |  |  | **log(OR)** | **95% CI^1^** | **p-value** |
| Age | -0.01 | -0.04, 0.01 | 0.3 |  | Age | 0.08 | -0.10, 0.26 | 0.4 |
| Sex |  |  |  |  | Sex |  |  |  |
| Male | — | — |  |  | Male | — | — |  |
| Female | 0.20 | -0.38, 0.79 | 0.5 |  | Female | 0.45 | -2.4, 3.3 | 0.8 |
| Alcohol regular history |  |  |  |  | Years of education | 0.03 | -0.56, 0.62 | 0.9 |
| No | — | — |  |  | Alcohol regular history |  |  |  |
| Yes | 0.15 | -0.51, 0.80 | 0.7 |  | No | — | — |  |
| Timepoints | -0.03 | -0.07, 0.02 | 0.2 |  | Yes | 4.7 | -2.8, 12 | 0.2 |
| Cigarettes smoke history |  |  |  |  | Timepoints | -0.77 | -1.5, -0.08 | 0.028* |
| No | — | — |  |  | Cigarettes smoke history |  |  |  |
| Yes | -0.10 | -0.65, 0.46 | 0.7 |  | No | — | — |  |
| *^1^CI = Confidence Interval*  **p < .05, ** p < .01, *** p < .001* | | | |  | Yes | 1.8 | -1.1, 4.8 | 0.2 |
|  |  |  |  |  | *^1^OR = Odds Ratio, CI = Confidence Interval*  **p < .05, ** p < .01, *** p < .001* | | | |
| **QUIP_any** | | | |  |  | | | |
|  | **log(OR)** | **95% CI^1^** | **p-value** |  |  |  |  |  |
| Age | 0.03 | -0.24, 0.31 | 0.8 |  |  |  |  |  |
| Sex |  |  |  |  |  |  |  |  |
| Male | — | — |  |  |  |  |  |  |
| Female | -0.40 | -5.4, 4.6 | 0.9 |  |  |  |  |  |
| Years of education | -0.08 | -0.94, 0.77 | 0.8 |  |  |  |  |  |
| Alcohol regular history |  |  |  |  |  |  |  |  |
| No | — | — |  |  |  |  |  |  |
| Yes | 0.30 | -6.2, 6.8 | 0.9 |  |  |  |  |  |
| Timepoints | -0.75 | -1.9, 0.43 | 0.2 |  |  |  |  |  |
| Cigarettes smoke history |  |  |  |  |  |  |  |  |
| No | — | — |  |  |  |  |  |  |
| Yes | 1.4 | -3.4, 6.3 | 0.6 |  |  |  |  |  |
| ^1^OR = Odds Ratio, CI = Confidence Interval  **p < .05, ** p < .01, *** p < .001* | | | |  |  | | | |
